# Kidney Function, Endothelial Activation and Atherosclerosis in Black and White Africans with Rheumatoid Arthritis

**DOI:** 10.1371/journal.pone.0121693

**Published:** 2015-03-25

**Authors:** Patrick H. Dessein, Hon-Chun Hsu, Linda Tsang, Aletta M. E. Millen, Angela J. Woodiwiss, Gavin R. Norton, Ahmed Solomon, Miguel A. Gonzalez-Gay

**Affiliations:** 1 Cardiovascular Pathophysiology and Genomics Research Unit, School of Physiology, Faculty of Health Sciences, University of the Witwatersrand, Johannesburg, South Africa; 2 Department of Nephrology, Milpark Hospital, Johannesburg, South Africa; 3 Department of Rheumatology, Charlotte Maxeke Johannesburg Academic Hospital, Faculty of Health Sciences, University of the Witwatersrand, Johannesburg, South Africa; 4 Epidemiology, Genetics and Atherosclerosis Research Group on Systemic Inflammatory Diseases, Rheumatology Division, Hospital Universitario Marques de Valdecilla, IDIVAL, Santander, Spain; National Cardiovascular Center Hospital, JAPAN

## Abstract

**Objective:**

To determine whether kidney function independently relates to endothelial activation and ultrasound determined carotid atherosclerosis in black and white Africans with rheumatoid arthritis (RA).

**Methods:**

We calculated the Jelliffe, 5 Cockcroft-Gault equations, Salazar-Corcoran, Modification of Diet in Renal Disease (MDRD) and Chronic Kidney Disease Epidemiology Collaboration (CKD-EPI) estimated glomerular filtration rate (EGFR) equations in 233 (112 black) RA patients.

**Results:**

The CKD-EPI eGFR was <90 ml/min/1.73m^2^ in 49.1% and 30.6% of black and white patients, respectively (odds ratio (95% confidence interval) = 2.19 (1.28–3.75), p = 0.004). EGFRs were overall consistently associated with monocyte chemoattractant protein-1 and angiopoietin 2 concentrations in white patients, and with carotid intima-media thickness and plaque in black participants. Amongst black patients, plaque prevalence was 36.7% and the area under the curve (AUC) of the receiver operating characteristic (ROC) curve was not associated with plaque presence for the MDRD equation (p = 0.3), whereas the respective relationship was significant or borderline significant (p = 0.003 to 0.08) and of similar extent (p>0.1 for comparisons of AUC (SE)) for the other 8 equations. Based on optimal eGFR cutoff values with sensitivities and specificities ranging from 42 to 60% and 70 to 91% respectively, as determined in ROC curve analysis, a low eGFR increased the odds ratio for plaque 2.2 to 4.0 fold.

**Conclusion:**

Reduced kidney function is independently associated with atherosclerosis and endothelial activation in black and white Africans with RA, respectively. CKD is highly prevalent in black Africans with RA. Apart from the MDRD, eGFR equations are useful in predicting carotid plaque presence, a coronary heart disease equivalent, amongst black African RA patients.

## Introduction

In 1974, Lindner and colleagues [1] reported that atherosclerotic coronary heart disease (CHD) risk is increased in patients on dialysis. However, dialyzed patients experience increased mortality due to sudden death and heart failure more frequently than from atherosclerotic CHD [2]. Endothelial cell dysfunction comprises a central mechanism in the genesis of the different cardiovascular dysfunction aspects in chronic kidney disease (CKD) [2]. Mild renal impairment elevates cardiovascular disease (CVD) risk [3].

Patients with RA sustain an increased risk of CVD [4,5]. Reduced kidney function development is enhanced in patients with RA compared to non-RA persons and increases the risk of cardiovascular events in RA [6,7]. The potential impact of impaired kidney function on atherogenic mechanisms including endothelial activation and atherosclerosis in RA requires elucidation.

Both traditional and nontraditional cardiovascular risk factors are associated with prevalent and incident CVD in RA [8]. Accordingly, currently reported recommendations on CVD risk stratification in RA include the use of multiple traditional risk factor assessment equations such as the Framingham score and the Systematic COronary Risk Evaluation score (SCORE) in combination with consideration of RA characteristics [9]. Nevertheless, up to 85% of white RA patients considered to be at moderate CVD risk according to the latter approach were reported to have carotid artery plaque [10], which represents a CHD equivalent [11,12]. Importantly in the present context, we recently found that both traditional cardiovascular risk factors and RA characteristics were related to atherosclerosis in white but consistently not in black Africans with RA, this despite a similar atherosclerosis burden amongst the 2 groups [13–15]. Taken together, these findings call for additional easily available and reliable CVD risk markers in white and even more so in black patients with RA.

Creatinine concentrations are unreliable and creatinine clearance is no longer recommended to estimate kidney function as timed urine collections are cumbersome and prone to error [16]. The iothalamate, EDTA, diethylene triamine pentaacetic acid or iohexol clearance most accurately estimate glomerular filtration rate (GFR) [16]. However, performing these investigations is expensive and complex and not recommended in routine clinical practice [16].

The initially reported Jelliffe estimated glomerular filtration (eGFR) equation in 1973 was a landmark in the assessment of kidney function [17]. Subsequently, a large series of other equations calculated from serum creatinine concentrations as well as age and sex with or without the inclusion of anthropometric measures or race were reported. Amongst these, the Chronic Kidney Disease Epidemiology Collaboration (CKD-EPI) is most recent and was most extensively validated [18]. Both The Modification of Diet in Renal disease (MDRD) [19] and Chronic Kidney Disease Epidemiology Collaboration (CKD-EPI) equations were also validated in non-RA black Africans [20]. To date, only the Cockcroft-Gault (C-G) actual body weight equation has been validated in a small cohort of white RA patients [21]. The MDRD was reported to be less accurate than the C-G actual body weight as a measure of creatinine clearance in RA [22]. A further complicating factor in patients with RA is that they experience excess body fat for a given body mass index (BMI) and reduced muscle mass [23], which can result in an overestimation of kidney function [24–26]. In this regard, the Salazar-Corcoran equation and the substitution of actual body weight by various other weight measures in the C-G equation can improve kidney function evaluation in persons with an altered adiposity status [24–26].

In view of these considerations, we evaluated the independent relations of 9 different eGFR equation values with endothelial activation and atherosclerosis in black and white Africans with RA. We further aimed at determining the accuracy of eGFR equation results in identifying patients with carotid plaque or high risk atherosclerosis.

## Patients and Methods

### Patients

The present study was conducted according to the principles outlined in the Helsinki declaration. The Human Research Ethics Committee (Medical) from the University of the Witwatersrand in Johannesburg, South Africa approved the protocol (approval number: M06-07-33). Participants gave informed, written consent.

This was a two-center based prospective study, comprising consecutive patients. Two hundred and thirty three African patients (112 black and 121 white) that met the 1987 American College of Rheumatology [27] and 2010 American College of Rheumatology/European League against Rheumatism (EULAR) criteria for RA [28] were enrolled at the Charlotte Maxeke Johannesburg (n = 129) and Milpark Hospital (n = 104) in Johannesburg. All invited participants agreed to participate. Data were missing in fewer than 5% of any of the recorded characteristics.

### Baseline characteristics

Were assessed baseline characteristics using previously reported methods (13–15). Briefly, we recorded demographic features, and anthropometric characteristics including height, weight and waist and hip circumference were measured employing standard approaches. The body mass index (BMI) was calculated and abdominal obesity and fat distribution were estimated by waist circumference and waist-hip ratio respectively. We recorded disease duration and rheumatoid factor status. Anti-cyclic citrullinated peptide antibody concentrations were not consistently evaluated and therefore not included in the analysis. Disease activity was assessed by the Clinical Disease Activity Index (CDAI) and the Disease Activity Score in 28 joints (DAS28). Extra-articular manifestations included the current or previously recorded (hospital record review) presence of pericarditis, pleuritis, Felty’s syndrome, cutaneous vasculitis, neuropathy, scleritis or episcleritis, retinal vasculitis, glomerulonephritis, vasculitis affecting other organs, amyloidosis, keratoconjunctivitis sicca, xerostomia, Sjogren’s syndrome, pulmonary fibrosis, bronchiolitis obliterans organizing pneumonia, cervical myelopathy, subcutaneous nodules and rheumatoid nodules in other locations. C-reactive protein concentrations were determined using immunoturbidimetric methods. Standard laboratory blood tests of erythrocyte sedimentation rate, lipids and glucose were performed.

We recorded current smoking status and systolic and diastolic blood pressure. Hypertension was defined as an average systolic blood pressure ≥140 or/and diastolic blood pressure ≥90 mmHg or/and current use of antihypertensive medications. Dyslipidemia was diagnosed when the atherogenic index, i.e. the cholesterol-HDL cholesterol ratio, was >4. Diabetes was identified when glucose lowering agents were used or the fasting plasma glucose was ≥7 mmol/l. The Framingham score was calculated using algorithms [12].

### Endothelial activation

We measured early endothelial activation molecule concentrations including those of soluble E-selectin, vascular cell adhesion molecule-1 (VCAM-1), ICAM-1and monocyte chemoattractant protein-1 (MCP-1), as well as angiopoietin 2 and using solid-phase sandwich ELISA (Quantikine®HS, R & D Systems, Inc., Minneapolis, MN, USA). Their lower detection limits were 0.009 ng/l, 0.6 ng/l, 0.096 ng/l, 5.0 pg/ml and 1.2 pg/ml respectively; their inter- and intra-assay coefficients of variation were 7.9 and 5.8, 7.0 and 3.1, 5.5 and 4.6, 5.7 and 5.8, and 8.9 and 5.9% respectively.

### Atherosclerosis

BAS (see acknowledgement) and AS performed the carotid artery ultrasound measurements in private and public healthcare patients, respectively. Both operators obtained images of at least 1cm length of the distal common carotid arteries for measurement of the intima-media thickness of the far wall from an optimal angle of incidence defined as the longitudinal angle of approach where both branches of the internal and external carotid artery are visualized simultaneously [29] and with high resolution B-mode ultrasound (Image Point, Hewlett Packard, Andover, MA, USA and SonoCalc IMT, Sonosite Inc, Bothell, Wash, USA used by BAS and AS, respectively) employing linear array 7.5 MHz probes. The details of the methodology used by BAS were reported previously [30]. The equipment used by AS involves the application of a unique semi-automated border detection program that was previously found to provide highly reproducible results [29]. The intima-media thicknesses in the left and right common carotid artery were measured and the cIMT was defined as the mean of these. Carotid artery plaque was defined as a focal structure that encroaches into the arterial lumen of at least 0.5 mm or 50% of the surrounding intima-media thickness value, or demonstrates a thickness of >1.5 mm as measured from the media-adventitia interface to the intima-lumen interface [31]. Both operators were blinded to the cardiovascular risk profiles of the patients. Repeat ultrasound examinations by both operators on 23 patients revealed Spearman correlations between repeat cIMT measurements of 0.983 and 0.956 for BAS and AS, respectively, and the correlation between measurements made by BAS and AS was 0.926. Both operators identified carotid artery bulb or/and internal carotid artery plaque in 11 of these 23 patients with full agreement.

### Kidney function

Serum creatinine concentrations were measured using the kinetic alkaline picrate method. In the 129 patients enrolled at the Charlotte Maxeke Johannesburg Academic Hospital, the Advia Chemistry systems (Siemens) was used with calibration traceable to isotope dilution mass spectrometry (IDMS); in the other 104 patients that were recruited at the Milpark Hospital, the Multiconstituent Calibrator (Architect) was employed which, at that time, was not traceable to IDMS. The non-IDMS and IDMS traceable values were inter-converted using the following equations: IDMS serum creatinine = (non-IDMS serum creatinine—0.067)/1.065 and non-IDMS serum creatinine = IDMS serum creatinine x 1.065 + 0.067 [32]. IDMS serum creatinine values were used in the CKD-EPI equation and non-IDMS results were employed in the other equations, as was defined when these equations were initially formulated [17–26]. Table 1 gives the 9 different eGFR equations [17–26] that were evaluated in the present study. The MDRD was calculated based on 4 variables [19]. The ethnicity factor as recommended in black Americans when calculating the MDRD and CKD-EPI was not applied in the present study as its use results in an overestimation of kidney function in black Africans living in our region [20].

**Table 1:** Estimated glomerular filtration rate equations.

Jelliffe	Men: (98–0.8 x (age—20)) / Scr; women: (98–0.8 x (age—20)) / Scr x 0.9
Cockcroft Gault ABW	Men: ((140—age) x ABW) / (Scr x 72); women: ((140—age) x ABW) / (Scr x 72) x 0.85
Cockcroft Gault IBW	Men: ((140—age) x IBW) / (Scr x 72); women: ((140—age) x IBW) / (Scr x 72) x 0.85; with IBW = 50 kg + (0.906 x (height-152.4)) in men and 45.5 kg + (0.906 x (height-152.4)) in women
Cockcroft Gault LBW	Men: ((140—age) x LBW) / (Scr x 72); women: ((140—age) x LBW) / (Scr x 72) x 0.85; with LBW = (9270 x ABW) / (6680 + (216 x BMI)) in men and (9270 x ABW) / (8780 + (244 x BMI)) in women
Cockcroft Gault ADBW	Men: ((140—age) x ADBW) / (Scr x 72); women: ((140—age) x ADBW) / (Scr x 72) x 0.85; with ADBW = IBW + (0.4 x (ABW—IBW))
Cockcroft Gault NBW	Men: (140—age) / Scr; women: (140—age) / Scr x 0.85
Salazar-Corcoran	Men: ((137—age x ((0.285 x weight) + (12.1 x height^2^))) / (51 x Scr); women: ((146—age x ((0.287 x weight) + (9.74 x height^2^))) / (60 x Scr)
MDRD	Men: 186 x Scr^−1.154^ x age^−0.203^; women: 186 x Scr^−1.154^ x age^−0.203^ 0.742
CKD-EPI	Women when Scr ≤0.7: 144 x (Scr / 0.7)^−0.329^ x 0.993^age^ and when Scr >0.7: 144 x (Scr / 0.7)^−1.209^ x 0.993^age^; men when Scr ≤0.9: 141 x (Scr 0.9) ^−0.411^ x 0.993^age^ and when Scr >0.9: 141 x (Scr / 0.9)^−1.209^ x 0.993^age^

Ideal body weight was calculated using the formula by Devine (34) with height measured in centimeters. In the Salazar-Corcoran equation, weight is expressed in kilogram and height in meter.

Scr = serum creatinine, AWB = actual body weight, IBW = ideal body weight, LBW = lean body Weight, ADBW = adjusted body weight, NBW = no body weight, MDRD = Modification of Diet in Renal Disease, CKD-EPI = Chronic Kidney Disease Epidemiology Collaboration.

### Data analysis

Dichotomous variables are expressed as proportions or percentages and continuous variables as mean (SD) or median (interquartile range) when non-normally distributed. Non-normally distributed characteristics were also logarithmically transformed prior to their inclusion in multivariable statistical analysis.

The recorded characteristics were compared between black and white patients using the Student t test, Mann-Whitney U or univariate logistic regression analysis as appropriate. To identify potential confounding variables in subsequent analysis, we assessed the associations of baseline characteristics with eGFR equations at p≤0.2 in demographic characteristic adjusted linear regression models.

The independent relationships of eGFR equations with endothelial activation molecule concentrations and cIMT were identified in confounder adjusted linear regression models; independent associations between eGFR equations and carotid plaque were evaluated in logistic regression models.

When consistent independent relations were found between eGFR equations and plaque, sensitivity versus false positive frequency (1-specificity) for predicting plaque presence with eGFR levels was analyzed employing receiver operator characteristic (ROC) curves. The predictive accuracy of eGFR equations was evaluated by the area under the curve (AUC). In this analysis, we accounted for potential confounding by traditional CVD risk factors and RA characteristics by including as a covariate the Framingham score, this with the application of the EULAR multiplier of 1.5 in patients with 2 of 3 criteria comprising of (1) a disease duration >10 years, (2) rheumatoid factor positivity, and (3) the presence of extraarticular manifestation(s) and thereby giving the modified (m) Framingham score (9). To determine the optimal cut-off values of eGFR values in predicting plaque presence, we calculated the Youden index using the following formula: sensitivity + specificity − 1, with the maximum obtained value corresponding to the optimal cut-off point [33]. Positive and negative predictive values were determined by applying Bayes’ theorem [34]. We then reassessed the associations of eGFRs with plaque using the obtained cut-off values in mFramingham score adjusted logistic regression models.

Statistical computations were made using the GB Stat program (Dynamic Microsystems, Inc, Silverspring, Maryland, USA) and SPSS software, version 21 (SPSS, Armonk, NY, USA). Significance was set at p<0.05.

## Results

### Baseline characteristics

Table 2 gives the baseline characteristics and their comparisons between those obtained in black and white patients with RA. As compared to whites, black participants were more often women, experienced a larger adiposity burden, more active and severe RA except for less frequent extraarticular manifestations and more prevalent hypertension and diabetes. The mean (SD) Framingham score was 6 (7) and 5 (7) in black and white patients, respectively. However, this characteristic was non-normally distributed with similar median (interquartile range) values of only 2 (1–5) and 3 (1–6) in the respective groups.

**Table 2:** Recorded characteristics in all 233 and 112 black and 121 white patients with RA.

**Demographic characteristics**	**All patients**	**Black**	**White**	**p**
Age (years)	57.1 (10.8)	55.7 (10.1)	58.3 (11.4)	0.06
Female sex	82.8	**88.4**	**77.7**	**0.03**
**Anthropometry**				
Body mass index (kg/m^2^)	27.4 (6.0)	**29.3 (6.6)**	**25.6 (6.6)**	**<0.0001**
Body mass index ≥25 (kg/m^2^)	58.8	**69.6**	**48.8**	**0.001**
Waist circumference (cm)	91 (13)	**93 (13)**	**89 (13)**	**0.01**
Waist/hip	0.86 (0.80–0.92)	0.85 (0.80–0.90)	0.87 (0.80–0.93)	0.2
**Cardiovascular agents**				
Antihypertensives	46.8	52.7	41.3	0.08
Statins	28.3	**19.6**	**36.4**	**0.005**
Ezetimibe	0.9	0	1.7	…
Oral glucose lowering agents	7.7	**12.5**	**3.3**	**0.01**
Insulin	1.7	1.8	1.7	0.9
**RA characteristics**				
Disease duration (years)	13.6 (9.3)	12.9 (9.2)	14.3 (9.4)	0.3
Rheumatoid factor positive	76.7	78.5	75.0	0.5
Clinical Disease Activity Index	7.2 (2.0–13.7)	**10.3 (4.0**–**15.5)**	**5.1 (0.7**–**11.7)**	**0.0001**
Disease Activity Score in 28 joints	3.9 (1.5)	**4.1 (1.3)**	**3.6 (1.6)**	**0.01**
Erythrocyte sedimentation rate (mm/hr)	12 (5–27)	**21 (9**–**31)**	**7 (3**–**14)**	**<0.0001**
C-reactive protein (mg/l)	5.1 (2.1–12.5)	**7.0 (4.0**–**13.8)**	**3.8 (1.5**–**10.6)**	**0.002**
Deformed joints (number)	6 (0–15)	**8 (3**–**14)**	**4 (0**–**17)**	**0.03**
Extraarticular manifestation(s)	7.7	**2.7**	**12.4**	**0.01**
Synthetic modifying agents				
Methotrexate	83.7	**90.2**	**77.7**	**0.01**
Chloroquine	66.1	**79.5**	**53.7**	**<0.0001**
Leflunomide	30.9	**22.3**	**38.8**	**0.007**
Sulphasalazine	18.5	**24.1**	**13.2**	**0.03**
Azathioprine	14.2	16.1	12.4	0.4
Tetracycline	12.0	10.7	13.2	0.6
Cyclophosphamide	3.0	5.4	0.8	0.08
Penicillamine	3.4	4.5	2.5	0.4
Number	2.4 (1.0)	**2.5 (1.0)**	**2.2 (0.9)**	**0.01**
Prednisone use	2.6	1.8	3.3	0.5
Tumor necrosis factor- blockade	3.9	0	7.4	…
NSAID	17.6	**7.1**	**27.3**	**0.0002**
**Conventional CV risk factors**				
Hypertension	57.5	**70.5**	**45.5**	**0.0001**
Systolic blood pressure (mmHg)	133 (21)	**139 (24)**	**128 (16)**	**0.0001**
Diastolic blood pressure (mmHg)	82 (12)	**86 (14)**	**79 (9)**	**<0.0001**
Total cholesterol (mM)	4.8 (1.0)	**4.7 (0.9)**	**5.0 (1.1)**	**0.02**
HDL cholesterol (mM)	1.5 (1.3–1.9)	1.5 (1.3–1.8)	1.6 (1.3–2.0)	0.07
LDL cholesterol (mM)	2.7 (0.9)	2.6 (0.8)	2.8 (0.9)	0.09
Triglycerides (mM)	1.0 (0.8–1.4)	1.0 (0.8–1.4)	1.0 (0.8–1.4)	0.6
Cholesterol/HDL cholesterol	3.2 (1.1)	3.3 (1.1)	3.2 (1.0)	0.5
Cholesterol/HDL cholesterol>4	19.7	23.9	16.0	0.1
Non-HDL cholesterol (mM)	3.2 (1.0)	3.1 (0.9)	3.3 (1.0)	0.2
Diabetes	12.5	**17.9**	**7.4**	**0.002**
Glucose (mM)	4.7 (4.4–5.2)	**4.9 (4.5**–**5.4)**	**4.7 (4.4**–**5.1)**	**0.02**
Smoking, current	6.9	3.6	10.0	0.07
Framingham score	2 (1–6)	2 (1–5)	3 (1–6)	0.4
**Endothelial activation**				
Early endothelial activation				
E-selectin (ng/ml)	38.6 (18.4)	**41.6 (19.8)**	**35.7 (16.5)**	**0.02**
VCAM-1 (ng/ml)	834 (664–1,048)	835 (696–1,043)	828 (635–1,033)	0.5
ICAM-1 (ng/ml)	277 (214–353)	**247 (173–317)**	**305 (251**–**385)**	**<0.0001**
MCP-1 (pg/ml)	427 (265–683)	**351 (223–679)**	**476 (334**–**684)**	**0.009**
Angiopoietin 2 (pg/ml)	2,502 (2,044–3,307)	**2,681 (2,232**–**3,566)**	**2,366 (1,924**–**3,130)**	**0.002**
**Carotid atherosclerosis**				
Intima-media thickness (mm)	0.709 (0.111)	0.703 (0.090))	0.715 (0.130)	0.4
Plaque	40.3	36.7	43.8	0.2
**Creatinine (mg/dl)**				
Non IDMS traceable	0.82 (0.72–0.96)	**0.87 (0.75**–**0.89)**	**0.79 (0.67**–**0.92)**	**0.006**
IDMS traceable	0.71 (0.61–0.84)	**0.76 (0.64**–**0.86)**	**0.68 (0.57**–**0.80)**	**0.006**
**EGFR equations**				
Jelliffe (ml/min)	79 (22)	**76 (19)**	**82 (24)**	**0.003**
C-G ACBW (ml/min)	91 (28)	90 (27)	92 (30)	0.2
C-G IBW (ml/min)	71 (23)	**65 (20)**	**77 (24)**	**<0.0001**
C-G ADBW (ml/min)	79 (23)	**75 (20)**	**83 (25)**	**0.0002**
C-G LBW (ml/min)	57 (17)	**54 (14)**	**59 (20)**	**0.001**
C-G NBW (ml/min)	91 (25)	**87 (22)**	**94 (28)**	**0.0003**
Salazar-Corcoran (ml/min)	89 (25)	**85 (22)**	**93 (28)**	**0.0003**
MDRD (ml/min/1.73 m^2^)	84 (24)	**78 (20)**	**89 (27)**	**0.0003**
CKD-EPI (ml/min/1.73 m^2^)	93 (17)	**90 (17)**	**95 (17)**	**0.0005**

Results are expressed as mean (SD), median (interquartile range) or proportion as appropriate.

Significant relations are shown in bold.

RA = rheumatoid arthritis, NSAID = non steroidal antiinflammatory agents, VCAM-1 = vascular adhesion molecule-1, ICAM-1 = intercellular adhesion molecule-1, MCP-1 = monocyte chemoattractant protein-1, IDMS = isotope dilution mass spectrometry, eGFR = estimated glomerular filtration rate, C-G = Cockcroft-Gault, AWB = actual body weight, IBW = ideal body weight, ADBW = adjusted body weight, LBW = lean body weight, NBW = no body weight, MDRD = Modification of Diet in Renal Disease, CKD-EPI = Chronic Kidney Disease Epidemiology Collaboration.

### Endothelial activation and atherosclerosis

E-selectin and angiopoietin 2 concentrations were larger and ICAM-1 and MCP-1 levels lower in black compared to white patients with RA. CIMT values and plaque frequency were similar in both groups.

### Kidney function

Table 2 also gives measures of kidney function in all, black and white patients. As previously reported in non-RA subjects [32], in all patients, IDMS creatinine concentrations were on average 13% smaller than those of non-IDMS creatinine. The mean (SD) C-G actual body weight and CKD-EPI eGFR values were similar (91 (28) ml/min versus 93 (17) ml/min/m^2^, p = 0.2). Application of the other equations produced consistently lower values (p<0.05 for comparisons with CKD-EPI eGFR), as also reported in non-RA subjects [24–26,35].

The non-IDMS and IDMS creatinine concentrations were higher in black compared to white patients. Kidney function as estimated by the C-G actual body weight equation was similar in both groups whereas application of each of the other 8 equations revealed reduced values in black compared to white patients. Taken together, as the C-G actual body weight equation overestimates kidney function in obese persons [24–26] and black patients experienced excess adiposity compared to whites (see Table 2), overall, these results indicate that renal function is impaired in the former compared to the latter group. In this regard also, CKD as defined by a CKD-EPI eGFR of <90 ml/min/m^2^ (3,18) was present in 49.1% and 30.6% of black and white RA patients, respectively; black population origin was associated with CKD (odds ratio (95% CI) for CKD = 2.19 (1.28–3.75), p = 0.004). Amongst all patients, the eGFR was mildly (60 to 89 ml/min/m^2^ (stage 2 CKD)) and moderately (30 to 59 ml/min/m^2^ (stage 3 CKD)) reduced in 84 (36.1%) and 8 (3.4%) (4 black and 4 white) RA participants, respectively.

### Baseline characteristics associated with kidney function

Associations between baseline characteristics and eGFR equations at p≤0.2 are shown in S1 Table. Demographic characteristics, adiposity indices, RA characteristics and traditional CVD risk factors were related to kidney function equations. The Framingham score was significantly associated with 7 of the eGFR equations and was included in subsequent analysis to account for potential confounding by traditional CVD risk factors.

### Kidney function and endothelial activation

Table 3 gives the independent relations of the eGFR equations with the endothelial activation markers MCP-1 and angiopoietin 2. In all patients, 4 eGFR equations were related to MCP-1 and 5 of them to angiopoietin 2. In stratified analysis, eGFR equations were not associated with endothelial activation amongst black Africans; by contrast, in whites, all equations were associated with MCP-1 concentrations and all except the Jelliffe, C-G no body weight and MDRD equation were related to angiopoietin 2 levels. Kidney function was unrelated to E-selectin, VCAM-1 and ICAM-1 concentrations (see S2 Table).

**Table 3:** EGFR formulas and endothelial activation in all, black, white, normal weight and obese patients with RA.

	**All patients**		**Black patients**		**White patients**	
	**MCP-1**	**Angiopoietin 2**	**MCP-1**	**Angiopoietin 2**	**MCP-1**	**Angiopoietin 2**
**EGFR equation**	**β (SE); p**	**β (SE); p**	**β (SE); p**	**β (SE); p**	**β (SE); p**	**β (SE); p**
Jelliffe	**−0.002 (0.001); 0.04**	−0.001 (0.001); 0.2	−0.001 (0.002); 0.6	0.001 (0.001); 0.4	**−0.003 (0.001); 0.02**	−0.001 (0.001); 0.2
C-G ACBW	−0.002 (0.001); 0.05	−0.001 (0.001); 0.05	−0.000 (0.002); 0.8	−0.000 (0.001); 0.8	**−0.003 (0.001); 0.01**	**−0.002 (0.001); 0.04**
C-G IBW	−0.002 (0.001); 0.07	**−0.001 (0.001); 0.02**	0.000 (0.002); 0.9	−0.000 (0.001); 0.6	**−0.003 (0.001); 0.009**	**−0.002 (0.001); 0.03**
C-G ADBW	−0.002 (0.001); 0.06	**−0.001 (0.001); 0.03**	−0.000 (0.002); 1.0	−0.000 (0.001); 0.7	**−0.003 (0.001); 0.009**	**−0.002 (0.001); 0.04**
C-G LBW	**−0.003 (0.001); 0.04**	**−0.002 (0.001); 0.006**	−0.000 (0.002); 0.9	−0.001 (0.001); 0.3	**−0.004 (0.001); 0.008**	**−0.003 (0.001); 0.01**
C-G NBW	**−0.002 (0.001); 0.04**	−0.001 (0.001); 0.1	−0.001 (0.002); 0.6	−0.000 (0.001); 0.6	**−0.002 (0.001); 0.02**	−0.001 (0.001); 0.2
Salazar-Corcoran	−0.002 (0.001); 0.06	**−0.001 (0.001); 0.04**	−0.000 (0.002); 1.0	−0.000 (0.001); 0.8	**−0.003 (0.001); 0.009**	**−0.002 (0.001); 0.04**
MDRD	−0.002 (0.001); 0.05	−0.001 (0.001); 0.2	−0.000 (0.002); 0.9	0.000 (0.001); 1.0	**−0.002 (0.001); 0.02**	−0.001 (0.001); 0.3
CKD-EPI	**−0.003 (0.001); 0.01**	**−0.002 (0.001); 0.04**	−0.002 (0.002); 0.4	−0.000 (0.001); 1.0	**−0.004 (0.002); 0.008**	**−0.003 (0.001); 0.04**

Data were analyzed in BMI, Framingham score, ethnicity, deformed joints, CDAI, chloroquine, leflunomide, penicillamine, prednisone and non-steroidal antiinflammatory agent use adjusted linear regression models.

Significant relations are shown in bold.

EGFR = estimated glomerular filtration rate, RA = rheumatoid arthritis, MCP-1 = monocyte chemoattractant protein-1, C-G = Cockcroft-Gault, AWB = actual body weight, IBW = ideal body weight, ADBW = adjusted body weight, LBW = lean body weight, NBW = no body weight, MDRD = Modification of Diet in Renal Disease, CKD-EPI = Chronic Kidney Disease Epidemiology Collaboration.

### Kidney function and atherosclerosis

Table 4 gives the independent relations of eGFR equations with atherosclerosis. In all patients, the Jelliffe, C-G ideal body weight, C-G no body weight, Salazar-Corcoran and CKD-EPI equations were associated with cIMT, and the Jelliffe, C-G no body weight and CKD-EPI equations with plaque. In stratified analysis, all except the MDRD equation were related to cIMT and all equations were associated with plaque in black patients; a 1 SD increase in eGFR was associated with an odds ratio of 0.34 (C-G actual body weight) to 0.45 (MDRD) for plaque. By contrast, no associations between eGFR equations and atherosclerosis were found in whites.

**Table 4:** Associations of EGFR with carotid intima-media thickness and plaque (per 1 SD increase in eGFR) in all, black and white patients with RA.

	**All patients**		**Black patients**		**White patients**	
	**CIMT**	**plaque**	**CIMT**	**plaque**	**CIMT**	**plaque**
**EGFR equation**	**β (SE); p**	**OR (95% CI); p**	**β (SE); p**	**OR (95% CI); p**	**β (SE); p**	**OR (95% CI); p**
Jelliffe	**−0.001 (0.000); 0.005**	**0.65 (0.47–0.89); 0.008**	**−0.002 (0.000); 0.002**	**0.38 (0.20–0.69); 0.001**	−0.001 (0.000); 0.3	0.88 (0.59–1.31); 0.5
C-G ACBW	−0.001(0.000); 0.06	0.73 (0.52–1.02); 0.07	**−0.001 (0.000); 0.006**	**0.34 (0.17–0.67); 0.001**	−0.000 (0.000); 0.5	1.04 (0.67–1.63); 0.8
C-G IBW	**−0.001(0.000); 0.04**	0.74 (0.54–1.01); 0.06	**−0.001 (0.000); 0.003**	**0.37 (0.20–0.70); 0.002**	−0.000 (0.000); 0.5	1.00 (0.98–1.02); 0.8
C-G ADBW	−0.001 (0.000); 0.05	0.75 (0.55–1.01); 0.06	**−0.001 (0.000); 0.004**	**0.38 (0.21–0.69); 0.001**	−0.000 (0.000); 0.5	1.05 (0.71–1.55); 0.8
C-G LBW	−0.001 (0.000); 0.1	0.84 (0.62–1.13); 0.8	**−0.002 (0.001); 0.01**	**0.39 (0.21–0.72); 0.002**	−0.000 (0.001); 0.6	1.21 (0.82–1.80); 0.3
C-G NBW	**−0.001 (0.000); 0.006**	**0.66 (0.48–0.90); 0.009**	**−0.001 (0.000); 0.001**	**0.39 (0.20–0.68); 0.001**	−0.000 (0.000); 0.3	0.91 (0.61–1.36); 0.6
Salazar-Corcoran	**−0.0001 (0.000); 0.04**	0.74 (0.55–1.00); 0.05	**−0.001 (0.000); 0.003**	**0.38 (0.21–0.70); 0.001**	−0.000 (0.000); 0.5	1.04(0.70–1.54); 0.8
MDRD	−0.000 (0.000); 0.3	0.94 (0.69–1.27); 0.7	−0.001 (0.000); 0.06	**0.45 (0.24–0.84); 0.01**	−0.000 (0.000); 1.0	1.42 (0.94–2.13); 0.09
CKD-EPI	**−0.001 (0.001); 0.005**	**0.68 (0.50–0.91); 0.01**	**−0.002 (0.000); 0.004**	**0.44 (0.26–0.73); 0.001**	−0.000 (0.001); 0.2	0.94 (0.61–1.45); 0.8

Data were analyzed in BMI, Framingham score, ethnicity, deformed joints, CDAI, chloroquine, leflunomide, penicillamine, prednisone and non-steroidal antiinflammatory agent use adjusted linear or logistic regression models for cIMT and plaque, respectively.

Significant relations are shown in bold.

EGFR = estimated glomerular filtration rate, SD = standard deviation, RA = rheumatoid arthritis, cIMT = carotid intima-media thickness, C-G = Cockcroft-Gault, AWB = actual body weight, IBW = ideal body weight, ADBW = adjusted body weight, LBW = lean body weight, NBW = no body Weight, MDRD = Modification of Diet in Renal Disease, CKD-EPI = Chronic Kidney Disease Epidemiology Collaboration.

**Fig 1:**
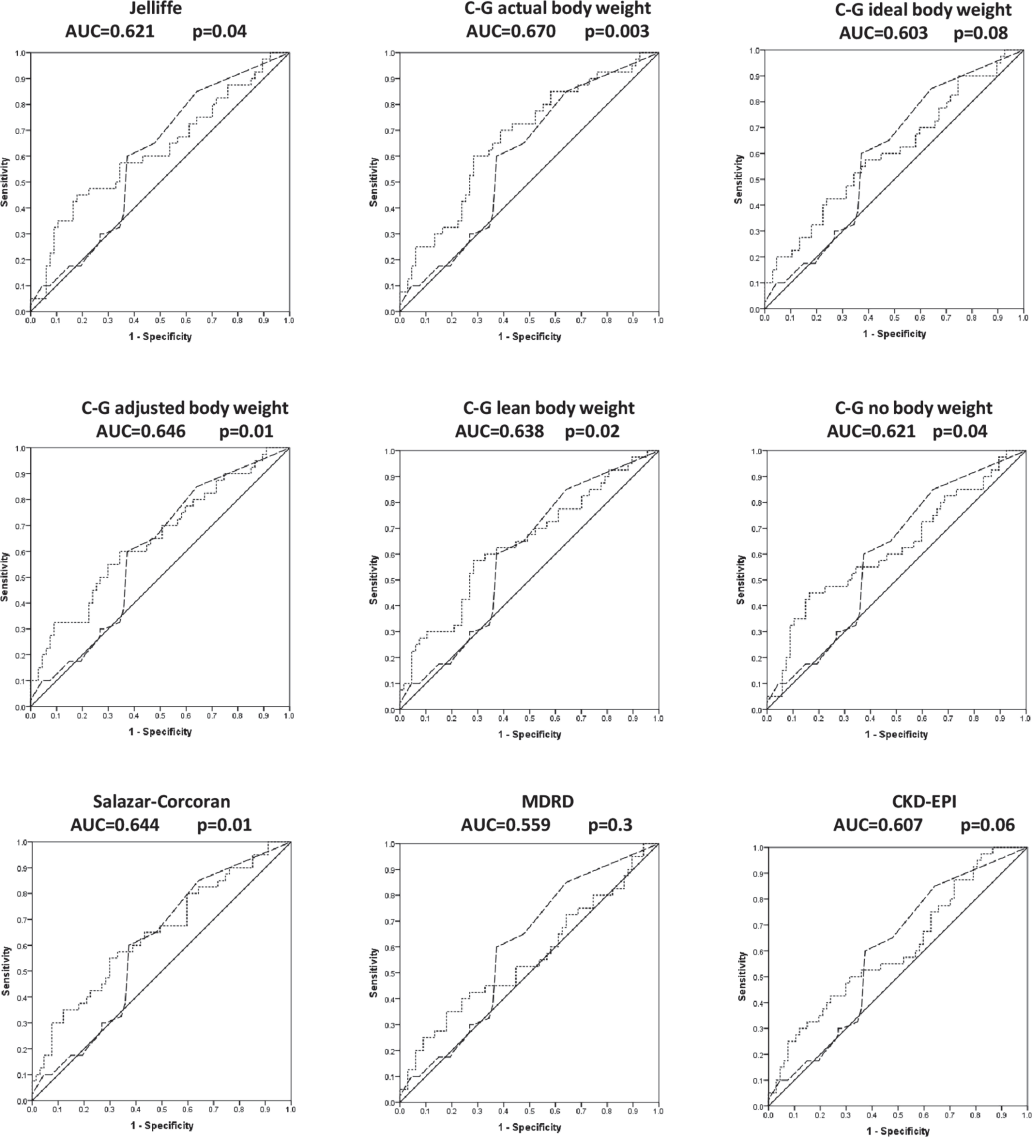
Receiver operator characteristic curves showing the accuracy of the eGFR equations (AUCs shown as dotted lines) in predicting plaque presence independent of the mFramingham score (AUCs shown as dashed lines). The *P* values given are for the AUC-plaque associations. The mFramingham score was consistently unrelated to plaque presence (AUC = 0.596 (*P* = 0.1) in each analysis). EGFR = estimated glomerular filtration rate; AUC = area under the curve; C-G = Cockcroft-Gault; MDRD = Modification of Diet in Renal Disease; CKD-EPI = Chronic Kidney Disease Epidemiology Collaboration.

As shown in Fig. 1, to estimate the accuracy of eGFR levels in the independent prediction of plaque presence amongst black patients with RA, we performed ROC curve analysis with the inclusion of the mFramingham score as a covariate. The mean (SD) and median (interquartile range) mFramingham scores were 6 (9) and 2 (1–8) in black African patients with RA. The area under the curve (AUC) of the ROC curve was not associated with plaque presence for the MDRD (p = 0.3) whereas the respective relation was significant or borderline significant and of similar extent (p>0.1 for comparisons of AUC (SE)) for the other 8 equations. The AUC of the ROC curve was consistently not associated with plaque presence for the mFramingham score.

To determine the optimal cut-off values for the eGFR equations in determining plaque presence we calculated the Youden index [33]. The obtained values and their corresponding sensitivity, specificity, and positive and negative predictive values as determined by applying Bayes’ theorem [34] are given in Table 5. EGFR values below these cut-off levels were significantly or borderline significantly associated with plaque with odds ratios ranging from 2.22 to 4.00.

**Table 5:** Optimal cut-off EGFR values in ROC curves with corresponding characteristics and associations with plaque in black patients with RA.

**EGFR**	**Cut-off value**	**Sensitivity (%)**	**Specificity (%)**	**PPV (%)**	**NPV (%)**	**OR (95% CI)** *	**p** *
Jelliffe	64	45	82	24	72	**3.61 (1.43–9.11)**	**0.007**
C-G ACBW	85	60	70	34	75	**3.36 (1.46–7.75)**	**0.004**
C-G IBW	54	42	88	22	72	2.22 (0.95–5.21)	0.07
C-G ADBW	68	55	81	29	75	**2.72 (1.19–6.20)**	**0.02**
C-G LBW	49	58	79	30	76	**3.31 (1.45–7.55)**	**0.005**
C-G NBW	73	45	84	24	72	**4.00 (1.57–10.17)**	**0.004**
Salazar-Corcoran	79	55	81	29	75	**2.72 (1.19–6.22)**	**0.02**
MDRD	64	35	91	18	70	2.49 (1.00–6.18)	0.05
CKD-EPI	82	42	91	21	73	2.22 (0.95–5.22)	0.07

Significant associations are shown in bold.

EGFR = estimated glomerular filtration rate, ROC = receiver operator characteristic, RA = rheumatoid arthritis, PPV = positive predictive value, NPV = negative predictive value, OR = odds ratio, CI = confidence interval, C-G = Cockcroft-Gault, AWB = actual body weight, IBW = ideal body weight, ADBW = adjusted body weight, LBW = lean body weight, NBW = no body weight, MDRD = Modification of Diet in Renal Disease, CKD-EPI = Chronic Kidney Disease Epidemiology Collaboration.

*Associations were assessed in modified Framingham score adjusted logistic regression models.

In 108 of the black patients with RA, IDMS traceable creatinine concentrations were obtained. As shown in S3 Table in a sensitivity analysis performed amongst these patients, the associations of eGFR equations with plaque were consistently numerically stronger with lower odds ratios than in the whole group (Table 4), irrespective of whether conversion of IDMS creatinine to non-IDMS creatinine results was performed (the first 8 equations) or not (the CKD-EPI equation). This suggests that the use of converted creatinine results in eGFR equations does not affect their associations with CVD risk in RA.

### Metabolic risk factors and kidney function in black compared to white patients with RA

In the present study, metabolic risk factors including not only BMI but also hypertension and diabetes were more adverse in black compared to white patients with RA (Table 2) and, at the same time, associated with kidney function (S1 Table). Both BMI and 5 of the eGFR equations as used in the present study contain body weight. Hence, as was previously reported in non-RA subjects, positive associations between body weight and kidney function as estimated by the respective equations is expected [36]. However, this does not accurately represent a ‘cardio-renal’ relationship [36]. As shown in S4 Table File after adjustment for metabolic risk in multivariable linear regression models, eGFR equations that did not contain anthropometric measures (Jelliffe, C-G no body weight, MDRD and CKD-EPI) no longer differed amongst both groups. BMI had a strong attenuating effect on the race-eGFR equation relation.

## Discussion

Although ∼80% of cardiovascular disease now occurs in low or middle income countries, data on cardiovascular risk management mostly originate in developed populations [13–15]. The present study revealed that reduced kidney function as assessed by previously reported eGFR equations for use in clinical settings, is independently associated with increased atherosclerosis in black Africans with RA. EGFR equations comprise the first reported easily and in fact routinely available cardiovascular risk marker shown to be useful in CVD risk stratification amongst RA patients from black African ancestry. Indeed, in ROC curve analysis, apart from the MDRD, eGFR equation results that fell below optimal cutoff values with sensitivities, specificities and positive and negative predictive values ranging from 42 to 60%, 70 to 91%, 21 to 34% and 72 to 76% respectively, increased the odds ratio for plaque 2.2 to 4 fold. The relatively high specificities and negative predictive values suggest that African black RA patients with non-reduction in eGFR are unlikely to have plaque, whereas the low positive predictive values are expected as the prevalence of plaque was, although clinically relevant, numerically low at 36.7%. Carotid plaque represents very high CVD risk that generally requires the preventive use of CVD drugs in both non-RA and RA subjects [8,11,12,37,38]. The AUC of the ROC curve for the C-G actual body weight-plaque relations was as large as those recently reported for rheumatoid factor and anti-cyclic citrullinated peptide-radiographic progression relation amongst patients with early RA [39]. Our results are also congruent with our reported finding that major traditional cardiovascular risk factors as well as previously investigated disease characteristics are not related to atherosclerosis in black Africans with RA, as the mFramingham was not related to plaque in these patients [13–15].

An independent association of eGFR equation values with the endothelial activation markers MCP-1 and angiopoietin 2 concentrations was found in white Africans with RA. Increased production of MCP-1 and angiopoietin 2 was reported and is linked to inflammation and implicated in the enhanced CVD risk amongst non-RA CKD patients [40–42]. Angiopoietin 2 concentrations also predict mortality in CKD [42]. Recently, angiopoietin 2 was shown to impact proliferation and apoptosis of cardiac endothelial cells and promote mesenchymal transition thereby leading to cardiac fibrosis [41] and capillary rarefaction. These processes increase the susceptibility to arrhythmias and ischemic injury in CKD [2,41].

The disparities in kidney function-CVD relations amongst black and white Africans with RA in the present investigation require further exploration. In this regard however, black Africans experienced markedly reduced kidney function with a 2.2 fold risk of CKD when compared to whites. Kidney function was reported to decrease more rapidly with age in black persons [43]. The most important causes of CKD are hypertension and diabetes [2,4]. Additionally, obesity is associated with glomerular hyperfiltration that mediates glomerular sclerosis [44,45]. This together with the impact of obesity on other CKD risk factors including metabolic parameters and renin angiotensin system and sympathetic nervous system activation engenders reduced kidney function over time in obese subjects (44,45). In this study, disparities in kidney function amongst black and white Africans with RA were explained by more adverse metabolic risk factor profiles in the former group, particularly obesity and to a lesser extent hypertension and diabetes. Interestingly, BMI but not hypertension and diabetes, was recently also shown to associate with kidney function reduction development in a predominantly white RA cohort [4]. Compared to the present investigation, kidney function was more impaired in previous studies that documented its impact on incident cardiovascular events in RA [6,7].

We assessed kidney function using 9 different equations [17–26]. The association of the obtained results with endothelial activation was consistent across these different measures amongst white RA patients. Overall, the same applied to the relations of the different equations with atherosclerosis in black patients with RA. Nevertheless, the AUC of the ROC curve for the MDRD equation was not associated with plaque presence amongst black patients with RA. Our results suggest that the application of any of the other 8 assessed equations as examined in the present study is more reliable and therefore preferable to the MDRD equation for CVD risk stratification amongst black RA patients.

Can a single EGFR equation be recommended in the management of patients with RA? Kidney function evaluation is important for drug dosing [46], CKD staging in delineating renoprotective intervention strategies to prevent end stage renal disease [16], and CVD risk stratification and management [3]. Whereas only the C-G actual body weight was previously validated in white patients with RA (21), the CKD-EPI equation is currently most recommended in CKD staging in non-RA subjects. The C-G equations have become the standard for drug dosing. Nevertheless, recent recommendations from the National Kidney Education Program suggest that both the C-G and CKD-EPI equations can be used for drug dosing [46]. In the present investigation, application of the C-G actual body weight and CKD-EPI equations produced equivalent eGFR values that were further similarly associated with atherosclerosis in black patients and endothelial activation in whites. Calculation of the CKD-EPI equation does not require information on body weight measurements, which is mostly not available to reporting laboratories. Taken together, although the CKD-EPI equation awaits validation in patients with RA, our results suggest that its recommended use in non-RA subjects can be extended to the RA population. Notably, anthropometric measures are also not include in the C-G no body weight equation and a recent meta-analysis showed that amongst C-G equations, the C-G no body weight equation most closely estimates creatinine clearance in non-RA patients [26].

A limitation of the present study is its cross-sectional design, thereby precluding drawing inferences on the direction of causality. Kidney function was defined by estimating equations. A large number of potential confounders were included in the models on plaque in Table 4. When we re-assessed the associations of kidney function with plaque using a summary score of traditional and non-traditional cardiovascular risk factors being the mFramingham score, as currently recommended for CVD risk stratification amongst patients with RA (9) and as a single covariate in the analysis, the results were consistent (Table 5).

### Perspectives

Impaired kidney function is frequent and should therefore be routinely ascertained in RA patients. A C-G ACBW equation result of <85 ml/min, C-G NBW of <73 ml/min or CKD-EPI of <82 ml/min comprises a useful surrogate marker of high risk subclinical atherosclerosis in black African patients with RA. This finding can help in determining the need for enhanced CVD risk stratification by carotid ultrasound [8,11,12] or, intensified risk management with cardiovascular drugs including statins in black African RA patients who have no access to cardiovascular imaging.

## Conclusions

CKD is twice as prevalent in black compared to white Africans with RA. Reduced kidney function is independently associated with atherosclerosis and endothelial activation in black and white Africans with RA, respectively. Apart from the MDRD, eGFR equations are useful in CVD risk stratification in black African RA patients.

## Supporting Information

S1 Table Baseline characteristics that were associated with estimated glomerular filtration rate equations at p≤0.2. (DOC)

S2 Table Associations of EGFR equations with early endothelial activation molecule concentrations in all and black and white patients. (DOC)

S3 Table Associations of EGFR with carotid intima-media thickness and plaque (per 1 SD increase in eGFR) in 108 black patients with IDMS traceable creatinine results. (DOC)

S4 Table EGFR equations without anthropometric measures in black compared to white RA patients after adjustment for metabolic risk. (DOC)

S1 Dataset (XLS)
