# Supplementary material for: Kidney Function, Endothelial Activation and Atherosclerosis in Black and White Africans with Rheumatoid Arthritis
Source: PLoS One. 2015 Mar 25;10(3):e0121693. doi: 10.1371/journal.pone.0121693 (PMC4373952; doi:10.1371/journal.pone.0121693)
Supplement: S1 Table — (DOC) [file pone.0121693.s001.doc]

**S1 Table.** Baseline characteristics that were associated with estimated glomerular filtration rate equations at p<0.2.

|  | **Jelliffe** | **CG ACBW** | **CG IBW** | **CG ADBW** | **CG LBW** | **CG NBW** | **Salazar-C** | **SMDRD** | **CKD-EPI** |
| --- | --- | --- | --- | --- | --- | --- | --- | --- | --- |
| **Characteristic** | **β**  **(SE);**  **p** | **β**  **(SE);**  **p** | **β**  **(SE);**  **p** | **β**  **(SE);**  **p** | **β**  **(SE);**  **p** | **β**  **(SE);**  **p** | **β**  **(SE);**  **p** | **β**  **(SE);**  **p** | **β**  **(SE);**  **p** |
| Age | **-1.006**  **(0.116);**  **<0.0001** | **-1.388**  **(0.149);**  **<0.0001** | **-1.119**  **(0.110);**  **<0.0001** | **-1.227**  **(0.113);**  **<0.0001** | **-0.855**  **(0.081);**  **<0.0001** | **-1.194**  **(0.134);**  **<0.0001** | **-1.304**  **(0.126);**  **<0.0001** | **-0.459**  **(0.143);**  **0.002** | **-0.781**  **(0.093);**  **<0.0001** |
| Female sex | 6.128  (3.329);  0.07 | **-8.958**  **(4.297);**  **0.04** | **-18.132**  **(3.150);**  **<0.0001** | **-14.463**  **(3.261);**  **<0.0001** | **-19.790**  **(2.329);**  **<0.0001** | 2.140  (3.841);  0.6 | **-12.475**  **(3.635);**  **0.0007** | -6.856  (4.122);  0.1 | -3.155  (2.671);  0.2 |
| Black race | **-9.254**  **(2.509);**  **0.0003** | -4.146  (3.239);  0.2 | **-12.640**  **(2.374);**  **<0.0001** | **-9.242**  **(2.458);**  **0.0002** | **-5.811**  **(1.756);**  **0.002** | **-10.623**  **(2.895);**  **0.0003** | **-9.908**  **(2.740);**  **0.0004** | **-11.472**  **(3.107);**  **0.0003** | **-7.094**  **(2.013);**  **0.0005** |
| BMI | **-0.695**  **(0.213);**  **0.001** | **2.375**  **(0.235);**  **<0.0001** | **-0.792**  **(0.201);**  **0.0001** | **0.475**  **(0.212);**  **0.02** | **0.541**  **(0.149);**  **0.0004** | **-0.804**  **(0.247);**  **0.001** | **0.481**  **(0.237);**  **0.04** | **-0.842**  **(0.266);**  **0.002** | **-0.439**  **(0.173);**  **0.01** |
| Waist | **-0.213**  **(0.095);**  **0.03** | **0.997**  **(0.105);**  **<0.0001** | -0.129  (0.090);  0.2 | **0.322**  **(0.092);**  **0.0005** | **0.300**  **(0.064);**  **<0.0001** | **-0.244**  **(0.110);**  **0.03** | **0.323**  **(0.103);**  **0.002** | **-0.242**  **(0.118);**  **0.04** | -0.147  (0.076);  0.05 |
| Log CDAI | 3.784  (2.704);  0.2 | 0.605  (3.506);  0.9 | 1.357  (2.569);  0.6 | 0.605  (3.506);  0.9 | 0.882  (1.899);  0.6 | 4.410  (3.120);  0.1 | 1.673  (2.964);  0.6 | 5.546  (3.343);  0.1 | 2.540  (2.172);  0.2 |
| Log deformed joints | -0.359  (2.424);  0.9 | **-8.630**  **(3.076);**  **0.005** | -0.896  (2.293);  0.7 | -3.991  (2.360);  0.09 | **-3.467**  **(1.681);**  **0.04** | -0.438  (2.797);  0.9 | -4.148  (2.633);  0.1 | -0.312  (3.002);  0.9 | -0.460  (1.944);  0.8 |
| Chloroquine use | **-8.887**  **(2.739);**  **0.001** | -6.489  (3.591);  0.07 | **-6.228**  **(2.619);**  **0.02** | **-6.332**  **(2.712);**  **0.02** | **-4.967**  **(1.932);**  **0.01** | **-10.353**  **(3.159);**  **0.001** | **-7.489**  **(3.019);**  **0.01** | **-10.929**  **(3.393);**  **0.01** | **-6.984**  **(2.199);**  **0.002** |
| Leflunomide use | **5.588**  **(2.736);**  **0.04** | 3.912  (3.554);  0.3 | 3.858  (2.600);  0.1 | 3.880  (2.693);  0.1 | 3.015  (1.921);  0.1 | **6.489**  **(3.156);**  **0.04** | 4.714  (2.999);  0.1 | 6.461  (3.392);  0.06 | 3.690  (2.201);  0.1 |
| Penicillamine  use | 10.180  (6.792);  0.1 | **25.633**  **(8.646);**  **0.003** | 6.821  (6.444);  0.3 | **14.346**  **(6.620);**  **0.03** | **11.193**  **(4.718);**  **0.02** | 11.746  (7.837);  0.1 | **15.851**  **(7.380);**  **0.03** | 12.860  (8.410);  0.1 | 7.120  (5.456);  0.2 |
| NSAID use | 4.180  (3.364);  0.2 | 7.504  (4.329);  0.08 | 3.698  (3.185);  0.2 | 5.220  (3.289);  0.1 | 3.604  (2.350);  0.1 | 4.697  (3.883);  0.2 | 5.974  (3.665);  0.1 | 5.502  (4.165);  0.2 | 2.744  (2.702);  0.3 |
| Prednisone use | **19.014**  **(7.752);**  **0.01** | 15.390  (10.087);  0.1 | **14.741**  **(7.368);**  **0.04** | 15.000  (7.630);  0.05 | 10.190  (5.454);  0.06 | **21.788**  **(8.946);**  **0.01** | **17.553**  **(8.497);**  **0.04** | **23.909**  **(9.596);**  **0.01** | 12.469  (6.246);  0.05 |
| Hypertension | **-6.246 (2.637);**  **0.02** | 5.446  (3.427);  0.1 | 4.846  (2.506);  0.05 | -0.729 (2.615);  0.8 | 0.471  (1.868);  0.8 | -**7.112 (3.044);**  **0.02** | -1.189 (2.914);  0.7 | **-7.718 (3.266);**  **0.02** | **-4.513 (2.121);**  **0.03** |
| Systolic BP | -0.076  (-0.062);  0.2 | **0.169**  **(0.079);**  **0.03** | -0.063  (0.058);  0.3 | 0.030  (0.061);  0.6 | 0.035  (0.043);  0.4 | -0.087  (0.071);  0.2 | 0.028  (0.068);  0.7 | -0.097  (0.076);  0.2 | -0.067  (0.049);  0.2 |
| Diastolic BP | -0.104  (0.107);  0.3 | **0.406**  **(0.136);**  **0.003** | -0.053  (0.101);  0.6 | 0.131  (0.105);  0.2 | 0.123  (0.074);  0.1 | -0.117  (0.123);  0.3 | 0.131  (0.117);  0.3 | -0.134  (0.132);  0.3 | -0.108  (0.086);  0.2 |

**S1 Table Continued.** Baseline characteristics that were associated with estimated glomerular filtration rate equations at *P* < 0.2.

|  | Jelliffe | CG ACBW | CG IBW | CG ADBW | CG LBW | CG NBW | Salazar-C | SMDRD | CKD-EPI |
| --- | --- | --- | --- | --- | --- | --- | --- | --- | --- |
| Characteristic | β  (SE);  p | β  (SE);  p | β  (SE);  p | β  (SE);  p | β  (SE);  p | β  (SE);  p | β  (SE);  p | β  (SE);  p | β  (SE);  P |
| HDL cholesterol* | 9.728  (9.827);  0.3 | -6.195  (12.707);  0.6 | 8.557  (9.303);  0.4 | 2.677  (9.648);  0.8 | 0.568  (6.892);  0.9 | 11.658  (11.337);  0.3 | 3.966  (10.752);  0.7 | 12.140  (12.170);  0.3 | 11.314  (7.865);  0.2 |
| Triglycerides* | -10.312  (6.009);  0.9 | 3.689  (7.804);  0.6 | -9.124  (5.691);  0.1 | -4.036  (35.920);  0.5 | -1.599  (4.231);  0.7 | -11.861 (6.934);  0.09 | -4.604  (6.598);  0.5 | -12.718  (7.442);  0.09 | -7.799  (4.824);  0.1 |
| Diabetes | -6.254  (3.790);  0.1 | 4.091  (4.914);  0.4 | **-8.394**  **(3.565);**  **0.02** | -3.400  (3.729);  0.4 | -1.682  (2.665);  0.5 | -7.343  (4.372);  0.09 | -3.782  (4.156);  0.4 | -7.525  (4.695);  0.1 | -4.813  (3.042);  0.1 |
| Glucose* | -13.212  (10.548);  0.2 | 14.923  (13.629);  0.3 | -17.187  (9.952);  0.09 | -4.435  (10.367);  0.7 | -0.975 (7.407);  0.9 | -15.550  (12.170);  0.2 | -4.929  (11.556);  0.7 | -18.546  (13.051);  0.2 | -6.992  (8.480);  0.4 |
| Smoking | *.083  (4.923);  0.07 | 3.172  (6.400);  0.6 | **10.271**  **(4.645);**  **0.03** | 7.439  (4.835);  0.1 | 5.425  (3.452);  0.1 | 10.808  (5.679);  0.06 | 8.577  (5.387);  0.1 | **12.717**  **(6.085);**  **0.04** | 6.255  (3.958);  0.1 |
| Framingham* | **-15.344**  **(2.886);**  **<0.0001** | **9.355**  **(3.927);**  **0.02** | **-6.319**  **(3.057);**  **0.04** | **-7.540**  **(3.162);**  **0.02** | -1.107  (2.404);  0.6 | **-16.100**  **(3.359);**  **<0.0001** | **-9.295**  **(3.433);**  **0.007** | -3.914  (3.319);  0.2 | **-8.527**  **(2.337);**  **0.0003** |

Associations were assessed in demographic characteristic adjusted linear multivariable regression models.

C-G = Cockcroft-Gault, AWB = actual body weight, IBW = ideal body weight, ADBW = adjusted body weight, LBW = lean body weight, NBW = no body weight, Salazar-C =

Salazar-Corcoran, MDRD = Modification of Diet in Renal Disease, CKD-EPI = Chronic Kidney Disease Epidemiology Collaboration, BMI = body mass index, log =

logarithmically transformed, CDAI = Clinical Disease Activity Index, NSAID = non steroidal antiinflammatory agents, BP = blood pressure, HDL = high density lipoprotein.
