# Supplementary material for: Kidney Function, Endothelial Activation and Atherosclerosis in Black and White Africans with Rheumatoid Arthritis
Source: PLoS One. 2015 Mar 25;10(3):e0121693. doi: 10.1371/journal.pone.0121693 (PMC4373952; doi:10.1371/journal.pone.0121693)
Supplement: S2 Table — (DOC) [file pone.0121693.s002.doc]

**S2 Table.** Associations of EGFR equations with early endothelial activation molecule concentrations in all and black and white patients.

|  | **All patients** | | | **Black patients** | | | **White patients** | | |
| --- | --- | --- | --- | --- | --- | --- | --- | --- | --- |
|  | **E-selectin** | **VCAM-1** | **ICAM-1** | **E-selectin** | **VCAM-1** | **ICAM-1** | **E-selectin** | **VCAM-1** | **ICAM-1** |
| **EGFR**  **equation** | **β**  **(SE);**  **p** | **β**  **(SE);**  **p** | **β**  **(SE);**  **p** | **β**  **(SE);**  **p** | **β**  **(SE);**  **p** | **β**  **(SE);**  **p** | **β**  **(SE);**  **p** | **β**  **(SE);**  **p** | **β**  **(SE);**  **p** |
| Jelliffe | -0.088 (0.067);  0.2 | -0.001 (0.001);  0.05 | -0.001 (0.001);  0.09 | 0.047 (0.119);  0.7 | -0.001 (0.001);  0.3 | -0.000 (0.001);  0.6 | -0.122 (0.082);  0.1 | -0.001 (0.001);  0.08 | -0.001 (0.001);  0.08 |
| CG ACBW | -0.043 (0.057);  0.4 | -0.001 (0.000);  0.08 | -0.001 (0.001);  0.07 | 0.007 (0.099);  0.9 | -0.001 (0.001);  0.2 | -0.001 (0.001);  0.4 | -0.036 (0.069);  0.6 | -0.001 (0.001);  0.2 | -0.001 (0.001);  0.1 |
| C-G IBW | -0.043 (0.063);  0.5 | -0.001 (0.001);  0.2 | -0.001 (0.001);  0.06 | 0.017 (0.113);  0.9 | -0.001 (0.001);  0.4 | -0.001 (0.001);  0.6 | -0.027 (0.076);  0.7 | -0.001 (0.001);  0.3 | -0.001 (0.001);  0.1 |
| C-G ADBW | -0.044 (0.061);  0.5 | -0.001 (0.000);  0.1 | -0.001 (0.001);  0.07 | 0.013 (0.108);  0.9 | -0.001 (0.001);  0.3 | -0.001 (0.001);  0.5 | -0.031 (0.074);  0.7 | -0.001 (0.001);  0.3 | -0.001 (0.001);  0.1 |
| C-G LBW | -0.069 (0.081);  0.4 | -0.001 (0.001);  0.2 | -0.001 (0.001);  0.1 | -0.028 (0.150);  0.9 | -0.001 (0.001);  0.2 | -0.001 (0.001);  0.6 | -0.042 (0.096);  0.7 | -0.001 (0.001;  0.3 | -0.001 (0.001);  0.2 |
| C-G NBW | -0.076 (0.058);  0.2 | -0.001 (0.000);  0.05 | -0.001 (0.001);  0.1 | 0.029 (0.102);  0.8 | -0.001 (0.001);  0.3 | -0.000 (0.001);  0.7 | -0.101 (0.070);  0.2 | -0.001 (0.001);  0.09 | -0.001 (0.001);  0.09 |
| Salazar-Corcoran | -0.045 (0.056);  0.4 | -0.001 (0.000);  0.1 | -0.001 (0.001);  0.07 | 0.017 (0.099);  0.9 | -0.001 (0.001);  0.2 | -0.001 (0.001);  0.5 | -0.040 (0.068);  0.6 | -0.001 (0.001);  0.2 | -0.001 (0.001);  0.1 |
| MDRD | -0.097 (0.059);  0.1 | -0.001 (0.000);  0.05 | -0.001 (0.001);  0.1 | -0.012 (0.110);  0.9 | -0.001 (0.001);  0.4 | -0.000 (0.001);0.7 | -0.114 (0.068);  0.1 | -0.001 (0.001);  0.07 | -0.001 (0.001);  0.08 |
| CKD-EPI | -0.069 (0.082);  0.4 | -0.001 (0.001);  0.09 | -0.001 (0.001);  0.2 | 0.029 (0.130);  0.8 | -0.001 (0.001);  0.1 | -0.000 (0.001);  0.9 | -0.097 (0.109);  0.4 | -0.001 (0.001);  0.2 | -0.001 (0.001);  0.2 |

Data were analyzed in BMI, Framingham score, ethnicity, deformed joints, CDAI, chloroquine, leflunomide, penicillamine, prednisone and non-steroidal

antiinflammatory agent use adjusted linear regression models.

Significant relations are shown in bold.

EGFR = estimated glomerular filtration rate, RA = rheumatoid arthritis, VCAM-1 = vascular adhesion molecule-1, ICAM-1 = intercellular adhesion

molecule-1, C-G = Cockroft-Gault, AWB = actual body weight, IBW = ideal body weight, ADBW = adjusted body weight, LBW = lean body weight, NBW

= no body weight, MDRD = Modification of Diet in Renal Disease, CKD-EPI = Chronic Kidney Disease Epidemiology Collaboration.
