# Supplementary material for: Kidney Function, Endothelial Activation and Atherosclerosis in Black and White Africans with Rheumatoid Arthritis
Source: PLoS One. 2015 Mar 25;10(3):e0121693. doi: 10.1371/journal.pone.0121693 (PMC4373952; doi:10.1371/journal.pone.0121693)
Supplement: S3 Table — (DOC) [file pone.0121693.s003.doc]

**S3 Table.** Associations of EGFR with carotid intima-media

thickness and plaque (per 1 SD increase in eGFR) in 108 black patients with IDMS

traceable creatinine results.

|  | **CIMT** |  | **Plaque** |  |
| --- | --- | --- | --- | --- |
| **EGFR equation** | **β (SE)** | **p** | **OR (95% CI)** | **p** |
| Jelliffe | **-0.002 (0.001)** | **0.004** | **0.34 (0.18-0.65)** | **0.001** |
| C-G ACBW | **-0.001 (0.001)** | **0.002** | **0.28 (0.14-0.60)** | **0.008** |
| C-G IBW | **-0.002 (0.001)** | **0.001** | **0.34 (0.17-0.66)** | **0.002** |
| C-G ADBW | **-0.002 (0.000)** | **0.001** | **0.33 (0.17-0.65)** | **0.001** |
| C-G LBW | **-0.002 (0.001)** | **0.003** | **0.35 (0.18-0.70)** | **0.002** |
| C-G NBW | **-0.002 (0.000)** | **0.0003** | **0.34 (0.18-0.65)** | **0.002** |
| Salazar-Corcoran | **-0.001 (0.000)** | **0.0008** | **0.33 (0.17-0.70)** | **0.001** |
| MDRD | **-0.001 (0.000)** | **0.02** | **0.40 (0.21-0.80)** | **0.003** |
| CKD-EPI | **-0.002 (0.000)** | **0.002** | **0.41 (0.24-0.71)** | **0.001** |

Data were analyzed in BMI, Framingham score, ethnicity, deformed joints, CDAI,

chloroquine, leflunomide, penicillamine and non-steroidal antiinflammatory agent

use adjusted linear or logistic regression models for cIMT and plaque, respectively.

Significant relations are shown in bold.

EGFR = estimated glomerular filtration rate, SD = standard deviation, IDMS =

isotope dilution mass spectrometry, RA = rheumatoid arthritis, cIMT= carotid

intima-media thickness, C-G = Cockroft-Gault, AWB = actual body weight, IBW =

ideal body weight, ADBW = adjusted body weight, LBW = lean body weight,

NBW = no body weight, MDRD = Modification of Diet in Renal Disease,

CKD-EPI = Chronic Kidney Disease Epidemiology Collaboration.
