# Supplementary material for: Kidney Function, Endothelial Activation and Atherosclerosis in Black and White Africans with Rheumatoid Arthritis
Source: PLoS One. 2015 Mar 25;10(3):e0121693. doi: 10.1371/journal.pone.0121693 (PMC4373952; doi:10.1371/journal.pone.0121693)
Supplement: S4 Table — (DOC) [file pone.0121693.s004.doc]

**S4 Table.** EGFR equations without anthropometric measures in

black compared to white RA patients after adjustment for metabolic risk.

| **EGFR equation** | **Black (n=112)** | **White (n=121)** | **p*** | **p^†^** | **p^§^** |
| --- | --- | --- | --- | --- | --- |
| Jelliffe | 76 (19) | 82 (24) | 0.2 | 0.5 | 0.6 |
| C-G NBW | 87 (22) | 94 (28) | 0.2 | 0.4 | 0.5 |
| MDRD | **78 (20)** | **89 (27)** | **0.01** | **0.04** | 0.05 |
| CKD-EPI | 90 (17) | 95 (17) | 0.08 | 0.3 | 0.3 |

Significant associations are shown in bold.

EGFR = estimated glomerular filtration rate, C-G = Cockcroft-Gault, NBW = no

body weight, MDRD = Modification of Diet in Renal Disease, CKD-EPI = Chronic

Kidney Disease Epidemiology Collaboration.

*adjusted for body mass index

^†^additionally adjusted for hypertension

^§^additionally adjusted for diabetes
